# Amelioration of Experimental Autoimmune Encephalomyelitis by Anatabine

**DOI:** 10.1371/journal.pone.0055392

**Published:** 2013-01-30

**Authors:** Daniel Paris, David Beaulieu-Abdelahad, Myles Mullan, Ghania Ait-Ghezala, Venkat Mathura, Corbin Bachmeier, Fiona Crawford, Michael J. Mullan

**Affiliations:** Roskamp Institute, Sarasota, Florida, United States of America; University of Muenster, Germany

## Abstract

Anatabine, a naturally occurring alkaloid, is becoming a commonly used human food supplement, taken for its claimed anti-inflammatory properties although this has not yet been reported in human clinical trials. We have previously shown that anatabine does display certain anti-inflammatory properties and readily crosses the blood-brain barrier suggesting it could represent an important compound for mitigating neuro-inflammatory conditions. The present study was designed to determine whether anatabine had beneficial effects on the development of experimental autoimmune encephalomyelitis (EAE) in mice and to precisely determine its underlying mechanism of action in this mouse model of multiple sclerosis (MS). We found that orally administered anatabine markedly suppressed neurological deficits associated with EAE. Analyses of cytokine production in the periphery of the animals revealed that anatabine significantly reduced Th1 and Th17 cytokines known to contribute to the development of EAE. Anatabine appears to significantly suppress STAT3 and p65 NFκB phosphorylation in the spleen and the brain of EAE mice. These two transcription factors regulate a large array of inflammatory genes including cytokines suggesting a mechanism by which anatabine antagonizes pro-inflammatory cytokine production. Additionally, we found that anatabine alleviated the infiltration of macrophages/microglia and astrogliosis and significantly prevented demyelination in the spinal cord of EAE mice. Altogether our data suggest that anatabine may be effective in the treatment of MS and should be piloted in clinical trials.

## Introduction

Multiple sclerosis (MS) is a chronic inflammatory demyelinating autoimmune disorder of the central nervous system (CNS). Clinical symptoms of MS not only include motor disabilities but also cognitive deficits [1] and increasing evidence indicates that axonal and neuronal injury are present both in the white and grey matter areas emphasizing a degenerative disease course [2], [3]. A variety of drugs are now approved for the treatment of MS. All of these drugs have potentially serious side effects and can suffer response failure during prolonged treatment [4], [5]. There is therefore a significant need to develop novel and safer medications to treat MS.

Anatabine is a minor tobacco alkaloid which is also present in plants of the Solanacea family including green tomatoes, peppers and eggplants. Our previous work with anatabine has demonstrated that this compound possesses anti-inflammatory properties. In particular, we have shown that anatabine inhibits nuclear factor kappa-light-chain-enhancer of activated B cells (NFκB) signaling and readily crosses the blood-brain barrier [6] suggesting it could represent a suitable agent to treat neuro-inflammatory disorders. More recently, we have shown that anatabine prevents the release of inflammatory cytokines induced by an intraperitoneal injection of lipopolysaccharide in mice by blocking the activation of NFκB and signal transducer and activator of transcription 3 (STAT3) [7]. Anatabine has also recently been shown to ameliorate experimental autoimmune thyroiditis by reducing inflammatory genes expression in the thyroid including cytokines [8]. The aim of the present study was to investigate the effect of anatabine in a mouse model of MS. Experimental autoimmune encephalomyelitis (EAE) is an established model of MS characterized by inflammation and neurodegeneration in the CNS [9] reproducing clinical and histopathological similarities to the human disease [10], and has been widely used to test potential therapies. Several methods have been developed to induce EAE, and in this study, we investigated whether anatabine was effective at ameliorating the clinical severity of EAE induced by an encephalitogenic injection of myelin oligodendrocyte glycoprotein 35–55 amino acid peptide (MOG_35–55_) in C57Bl/6 mice. MOG induced EAE results in prevalent axonal degeneration and somatic neuronal loss [11], demyelination, motor disabilities as well as cognitive impairment associated with neuroinflammation [12]. Our study demonstrates that anatabine is effective at ameliorating the development of EAE. Anatabine appears to inhibit the release of proinflammatory cytokines induced during EAE and to prevent p65 NFκB and STAT3 phosphorylation in the CNS. These effects are accompanied by reduced microgliosis, astrogliosis and demyelination in the spinal cord of EAE mice. Other studies suggest that the plasma levels of anatabine that produce these effects in mice are attainable in humans without prohibitive side effects (Dr. Mullan, personal communication). Collectively, our data suggest that anatabine may be effective in the treatment of MS.

## Methods

### EAE Induction and anatabine treatment

All experiments involving mice were approved by the Institutional Animal Care and Use Committee of the Roskamp Institute (IACUC protocol # R35) and were performed in the Association for Assessment and Accreditation of Laboratory Animal Care International (AAALAC) accredited vivarium of the Roskamp Institute. C57BL/6J female mice were purchased from the Jackson Laboratories (ME, USA). The mice were 10 week-old at the beginning of the study. EAE was induced on day 1 by a subcutaneous injection of 300 µg of myelin oligodendrocyte glycoprotein (MOG_35–55_) peptide (AnaSpec, CA, USA) emulsified with complete Freud's adjuvant (Difco Laboratories/Beckton Dickinson, NJ, USA) containing 500 µg of heat-killed Mycobacterium tuberculosis H37 Ra (Difco Laboratories/Beckton Dickinson, NJ, USA) followed by an intraperitoneal injection of 300 ng of Pertussis toxin (Tocris, R&D Systems, MN, USA) immediately and 48 hours later. Anatabine was provided by Rock Creek Pharmaceuticals (MA, USA). On day 1, mice were randomized into a placebo group (n = 15) receiving regular drinking water (EAE placebo) and into an anatabine treatment group (n = 14) receiving anatabine in their drinking water (80 mg of anatabine per liter of drinking water (pH adjusted to 7.5 with HCl)) (EAE Anatabine) corresponding to an oral drug uptake of approximately 20 mg/Kg of body weight/day. In addition, a group of control non-immunized mice (n = 6) receiving regular drinking water was included in the study. After EAE induction, mice were monitored everyday using the following arbitrary scale: 0, no symptom; 1, flaccid tail; 2, hindlimb weakness or abnormal gait; 3, complete hindlimb paralysis; 4, moribund or deceased. On day 16, animals were humanely euthanatized and their serum, spleen, one brain hemisphere (without the cerebellum) were snap frozen in liquid nitrogen whereas the spinal cord and the other brain hemisphere were fixed in 4% paraformaldehyde at 4°C.

### Measurement of cytokines and anti-MOG antibody titer

Serum cytokines (IL-1β, IL-6, IL-10, IL-17A, IFN-γ, TNF-α) were evaluated using a Bio-plex Mouse cytokine panel with the Bio-Plex Suspension Array System (Biorad, CA, USA) according to the manufacturer's instructions. Spleen homogenates were obtained by sonicating the tissues in ice cold MPER reagent (Pierce Biotechnology, Rockford, IL, USA) containing 1X of Halt™ protease and phosphatase inhibitor cocktail (Thermo Scientist, IL, USA). Homogenates were centrifuged at 14,000 rpm for 30 minutes at 4°C and supernatants collected. IL -1β, IL-6, IFN-γ, TNF-α and IL-17 were evaluated in spleen supernatants using commercially available ELISAs (Life Technologies, NY, USA). Protein concentrations were quantified in spleen supernatants using the BCA method (Biorad, CA, USA) and results calculated in pg of cytokine/mg of protein and expressed as a% of the cytokine values observed in control non immunized mice. The titer of anti-MOG antibodies was quantified in the serum of the mice using a Sensolyte Anti-Mouse MOG_35–55_ IgG ELISA kit following the manufacturer's recommendations (AnaSpect, CA, USA).

### Western-blots

Western-blot experiments were carried out for the quantification of phosphorylated STAT3 and p65 NFκB using spleen and brain supernatants prepared as described above. Tissue supernatants were denatured by boiling in Laemmli buffer (Bio-Rad, CA, USA) and resolved onto 4–20% gradient polyacrylamide gels (Bio-Rad, CA, USA). After electrotransfering onto polyvinylidene difluoride membranes, western-blots were immunoprobed with an anti-actin antibody (Chemicon, CA, USA) used as a reference antibody to quantify the amount of proteins electrotransferred, with a phospho-STAT3 (Tyr705) antibody (Cell Signaling Technology Inc, MA, USA) and with a phospho-NFκB p65 (Ser536) antibody (Cell Signaling Technology Inc, MA, USA). Phospho-STAT3/actin and phospho-NFκB p65/actin signal intensity ratios were quantified by chemiluminescence imaging with the ChemiDocTM XRS (Bio-Rad, CA, USA).

### Pathological examinations

Mice were humanely euthanatized and brain and spinal cord immersed in 4% paraformaldehyde for 48 hours at 4°C and paraffin embedded. Six micrometer thick (6 µm) sagittal sections from embedded blocks of the brain were cut to include the cortex, hipocampal area and medulla and 6 µm thick longitudinal sections of the spinal cord were cut to include the majority of the length of the spinal cord (from cervical to lumbar regions), containing both gray and white matter. Prior to staining, sections were deparaffinized in xylene (2×5 min) and hydrated in graded ethanol (2×5 min in 100%, 5 min in 85%, 5 min 70%) to distilled water and finally rinsed in PBS. Spinal cord sections were stained with Hematoxylin/Eosin (Sigma, MO, USA) and with Luxol fast blue (American Mastertech, CA, USA) following the manufacturer's recommendations to assess cell infiltration and demyelination, respectively. Four to five different sections per mouse were analyzed under light microscopy (400X magnification). At least 10 randomly distributed 40X fields within the white matter of the spinal cord were captured for each section. The areas covered by the Luxol fast blue stain were quantified using Image-Pro Plus software (Media Cybernetics, MD, USA) and expressed as a percentage of the total area of the white matter examined (Luxol fast blue burden). An average value of the Luxol fast blue burden was determined for each mouse.

For immunohistochemistry, endogenous peroxidase activity was quenched with a 20-min-H_2_O_2_ treatment (0.3% in distilled water) and after being rinsed with PBS, sections were incubated with blocking buffer (Protein Block Serum-free, Dako, CA, USA) for 45 min. The following primary antibodies were diluted in Dako antibody diluent (CA, USA): 1∶300 dilution of Phospho-STAT3 rabbit antibody (Y705)(D3A7)(Cell Signaling Technology Inc, MA, USA); 1∶10,000 dilution of anti-Glial Fibrillary Acidic Protein (GFAP) rabbit antibody (Dako, CA, USA); 1∶1,000 dilution of anti-Iba1 (ionized calcium binding adaptor molecule 1) goat antibody (Abcam, MA, USA). The diluted antibodies were applied onto the sections overnight at 4°C and were detected using Vectastain ABC (avidin-biotin-peroxidase complex) Elite kits (Vector Laboratories, CA, USA). Phospho-STAT3 and Iba1 labeling were revealed using a 3,3’-diaminobenzidine (DAB)/Peroxidase Substrate kit (Vector laboratories, CA, USA). GFAP immunostaining was revealed using the nickel/DAB procedure following the manufacturer's recommendations (Vector laboratories, CA, USA). For each mouse, 4 to 5 immunostained sections of the brain and spinal cord were used to perform the quantification of GFAP and Iba1 burden. Randomly distributed digital pictures were taken at a 400X magnification by an experimenter unaware of the treatment conditions. Four to five non-overlapping pictures were taken in the cortex and medulla whereas the entire hipocampal area was analyzed for each brain section. At least 10 randomly distributed pictures along the entire length of the spinal cord were captured for each section of the spinal cord. The stained areas within particular regions of the brain (hippocampus, cortex, medulla) and within the white and grey matter of the spinal cord were quantified using Image-Pro Plus software (Media Cybernetics, MD, USA). An average value of GFAP and Iba1 burden was calculated for each individual mice and expressed as a percentage of the total brain area examined. Following the phospho-STAT3 immunostaining, the average number of phospho-STAT3 immunopositive cells was quantified in 40X fields of the cortex and the spinal cord (white matter) using four to five sections for each mice and Image-Pro Plus software (Media Cybernetics, MD, USA). An average number of phospho-STAT3 immunopositive cells per 40X microscopic field was determined for each mouse and expressed as a percentage of STAT3 immunopositive cells observed in EAE placebo mice.

### Statistical analyses

Results are expressed as the mean ± SEM. Statistical analyses were performed using SPSS V12.0.1 for Windows. Data were examined for assumption of normality using the Shapiro-Wilk statistic and for homogeneity of variance using the Levene's test. Statistical significance was determined by Student's *t*-test, univariate or repeated measures analysis of variance (ANOVA) where appropriate followed by post-hoc comparisons with Bonferroni corrections. For data not satisfying assumptions of normality and homogeneity of variance, a nonparametric Mann-Whitney test was used. P-values <0.05 were considered significant.

## Results

### Effects of anatabine on the clinical progression of EAE

**Figure 1:**
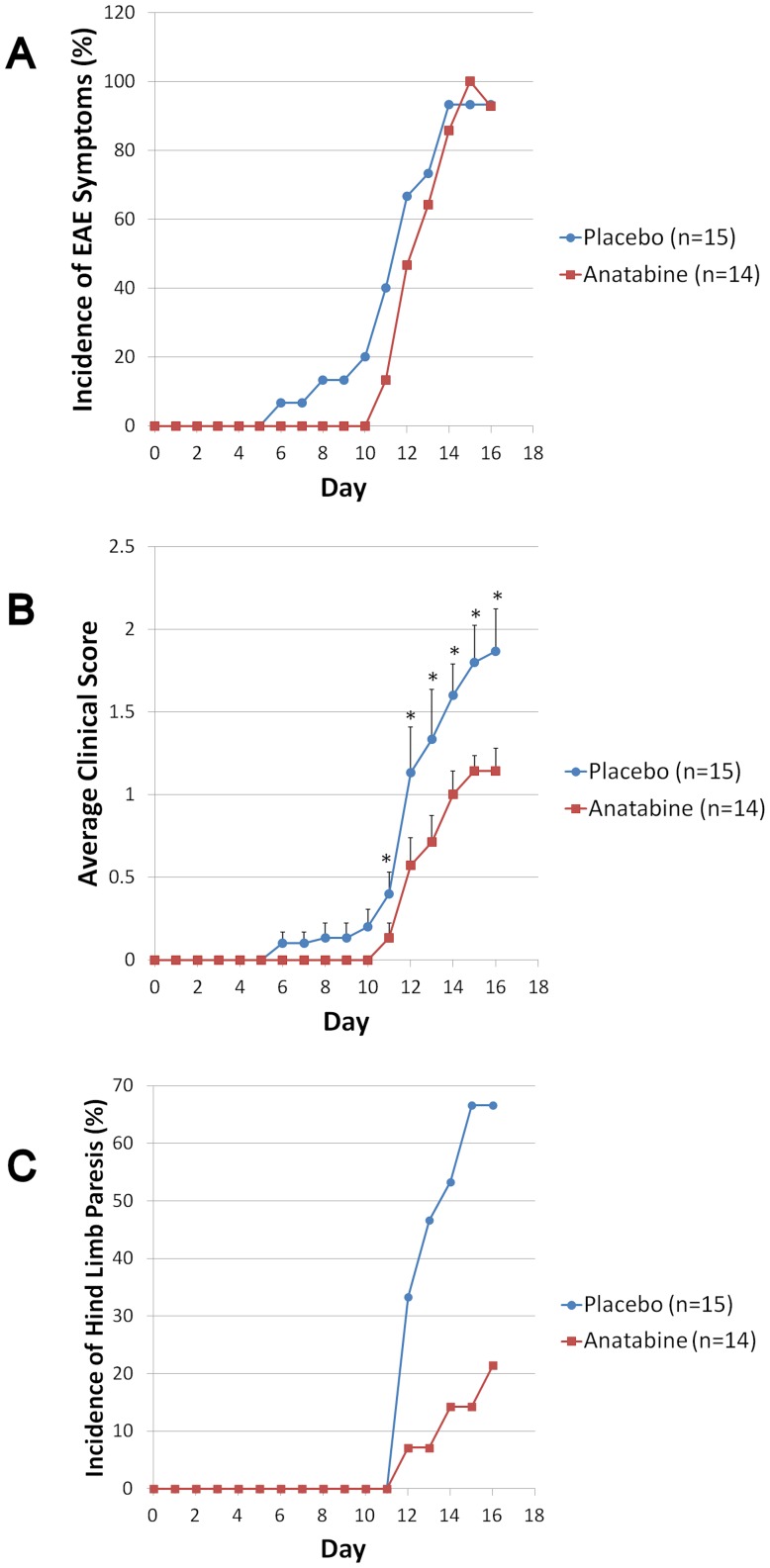
Treatment with anatabine ameliorates the clinical course of EAE. C57BL/6 mice were treated with 20 mg/Kg of body weight/day of anatabine directly dissolved in their drinking water or received regular drinking water (placebo) starting on the first day of the immunization (day 1). Mice were monitored daily for clinical disease and scored for clinical symptoms. A) Incidence of clinical symptoms of EAE in placebo and anatabine treated mice. MOG35–55 immunized mice receiving regular drinking water (placebo) develops clinical symptoms of EAE from day 6 after immunization whereas anatabine treated mice were present at day 6 following the immunization in placebo mice whereas the first incidence of EAE clinical symptoms was delayed to day 11 in anatabine treated mice. B) Anatabine reduces the clinical severity of EAE. Data for each point represents the average disease symptom score ± SEM. ANOVA reveals a significant main effect of anatabine (P<0.001) and time (P<0.001) as well as a significant interactive term between them (P<0.002) on the clinical severity of EAE. Post-hoc comparisons show statistically significant differences between the average clinical severity of EAE in the placebo and the anatabine treatment group from day 11 post-immunization to day 16 (* P<0.03). C) Anatabine suppresses the incidence of hind limb weakness/paralysis in EAE. By day 16 following the MOG immunization approximately 70% of the mice in the placebo group present hind limb weakness or complete paralysis whereas only 20% of the mice in the anatabine treatment group develop such symptoms.

**Figure 2:**
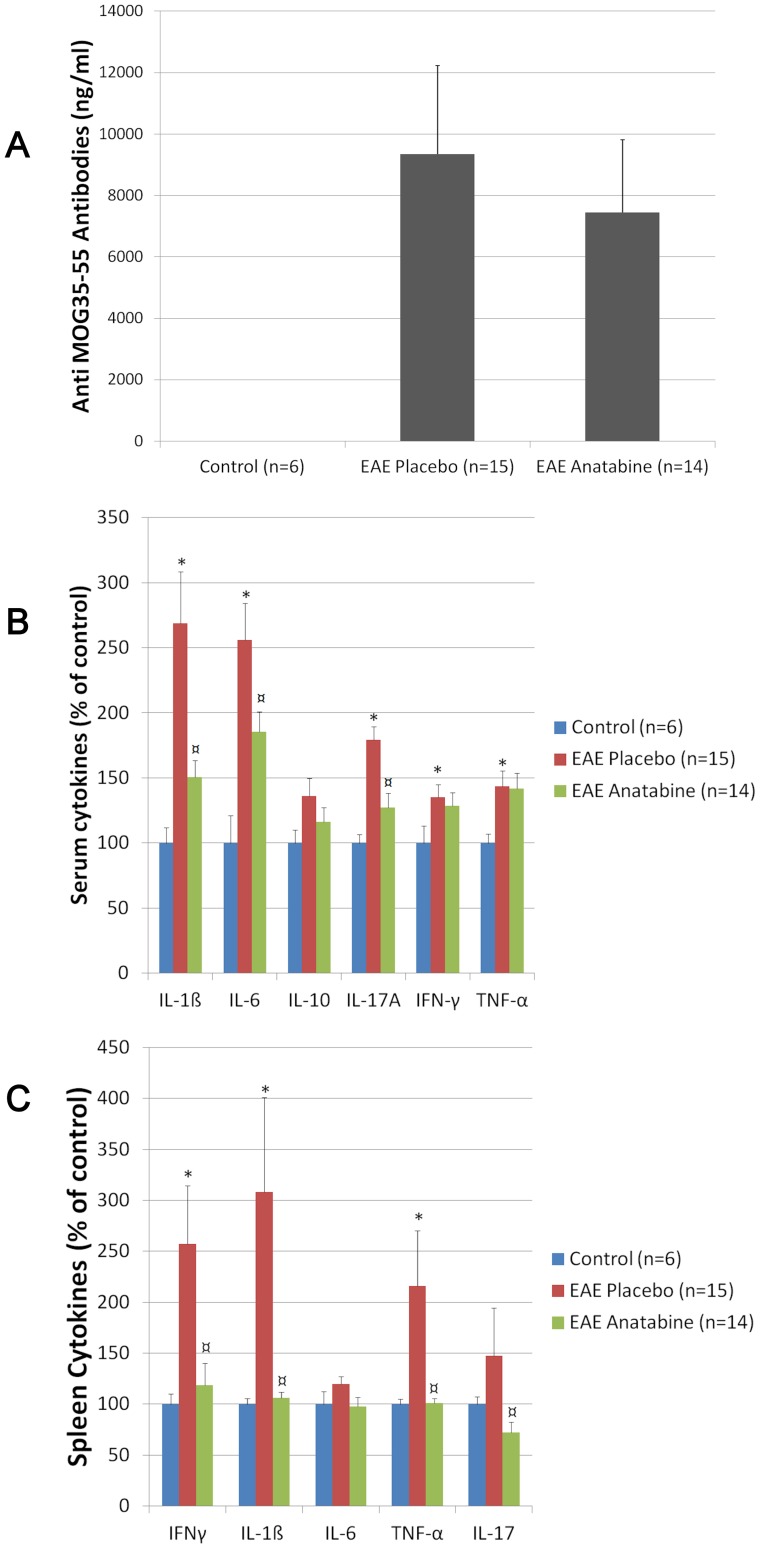
Administration of anatabine inhibits pro-inflammatory cytokines but does not affect the titer of circulating anti-MOG antibodies. A) Average amount of anti-MOG antibodies measured by ELISA in the serum of control non-immunized mice, EAE placebo and EAE anatabine treated mice at day 16 following the MOG immunization. ANOVA reveals a significant main effect of the MOG immunization on the titer of anti-MOG antibodies (P<0.001) but no significant main effect of anatabine (P = 0.617). B) Anatabine inhibits the production of pro-inflammatory cytokines in the serum of EAE mice. A significant elevation of TNF-α (Mann-Whitney U = 16.5, Z = −2.22, P = 0.026), IL-1β (Mann-Whitney U = 0, Z = −3.506, P<0.001), IL-6 (Mann-Whitney U = 3, Z = −3.271, P<0.001), IFN-γ (Mann-Whitney U = 16.5, Z = −2.219, P = 0.026) and IL-17 (Mann-Whitney U = 7, Z = −2.958, P = 0.003) was observed in the serum of EAE mice (placebo) compared to control non-immunized mice. Anatabine significantly prevented the production of IL-1β (Mann-Whitney U = 44, Z = −2.666, P = 0.008), IL-6 (Mann-Whitney U = 59, Z = −2.008, P = 0.045) and IL-17 (Mann-Whitney U = 47, Z = −2.531, P = 0.011) in the serum of EAE mice. C) Anatabine suppresses pro-inflammatory cytokine production in the spleen of EAE mice. A statistically significant elevation of TNF-α (Mann-Whitney U = 3, Z = −3.270, P = 0.001) and IL-1β (Mann-Whitney U = 0, Z = −3.503, P<0.001) was observed in the spleen of EAE placebo mice compared to control non-immunized mice. A significant decrease in TNF-α (Mann-Whitney U = 22, Z = −3.622, P<0.001), IL-1β (Mann-Whitney U = 9, Z = −4.19, P<0.001) and IL-17 (Mann-Whitney U = 55, Z = −1.976, P<0.05) was observed in the spleen of anatabine treated EAE mice compared to placebo EAE mice. (* P<0.05 for EAE placebo mice vs control non-immunized mice; ¤ P<0.05 for EAE placebo mice vs EAE anatabine treated mice).

In the present study, we used a MOG induced EAE model because it closely resembles many characteristics of MS such as T-cell mediated auto-inflammation in the CNS, axonal injury and demyelination. The axonal damage in EAE mice leads to well defined clinical signs such as tail paralysis, hind-limb weakness and paralysis. Anatabine was delivered in the drinking water of the animals at a dosage of 20 mg/Kg of body weight/Day from the time of immunization. This particular dosage was selected from a previous study showing that this dosage is efficient at lowering brain cytokine levels in a mouse model of Alzheimer's disease displaying chronic neuroinflammation [7]. Fig. 1 shows that nearly 100% of the mice immunized against murine MOG_35–55_ developed clinical signs of EAE both in the placebo and anatabine treatment group. Clinical signs of EAE became apparent by day 6 following the immunization in the placebo group but were delayed to day 11 in the anatabine treatment group (Fig. 1A). The average clinical severity of EAE was also significantly reduced by the anatabine treatment (Fig. 1B). Importantly, approximately 70% of the mice in the placebo group developed hind-limb weakness or paralysis compared to only 20% in the anatabine treatment group (Fig. 1C) showing that the mice treated with anatabine displayed significantly milder disease symptoms than the placebo group. We euthanatized the mice at day 16 following the immunization for pathological and biochemical evaluations because it has been shown that the peak of EAE disease severity is generally reached around day 16 in similar MOG immunization models [12], [13]. We first assessed the titer of circulating antibodies reacting against murine MOG_35–55_ in the serum of control non-immunized mice, EAE placebo and EAE anatabine treated mice. As expected MOG immunized animals display an elevated titer of anti-MOG antibodies compared to control non-immunized mice (Fig. 2A). We did not observe a difference between the titer of anti-MOG antibodies in the serum of EAE placebo and EAE mice treated with anatabine (Fig. 2A).

### Effect of anatabine on peripheral cytokine levels in EAE mice

We next evaluated the impact of anatabine on the expression of cytokines important in the development of EAE such as IL-1β, IFN-γ, TNF-α, IL-6 and IL-17A. We found that IL-1β, IFN-γ, TNF-α, IL-6 and IL-17A were significantly elevated in the serum of EAE mice compared to control non-immunized mice. However, anatabine significantly suppressed the production of IL-1β, IL-6 and IL-17A in the serum of EAE mice (Fig. 2B) but did not significantly impact IFN-γ and TNF-α. We did not observe a significant effect of EAE or anatabine on circulating IL-10 levels. We also evaluated the impact of EAE and anatabine on cytokine production in the spleen of the animals at day 16 post MOG immunization. A significant elevation of IL-1β, INF-γ and TNF-α was observed in the spleen of EAE mice compared to control non immunized animals. Anatabine appears to fully suppress IL-1β, INF-γ and TNF-α elevation in the spleen of EAE mice (Fig. 2C). We did not observe a significant effect of EAE on IL-6 or IL-17 production in the spleen of the animals. However, a significant reduction in IL-17 was observed in the spleen of EAE anatabine treated mice compared to EAE placebo mice (Fig. 2C).

### Effects of EAE and anatabine on STAT3 and p65 NFκB phosphorylation in the spleen and the brain

**Figure 3:**
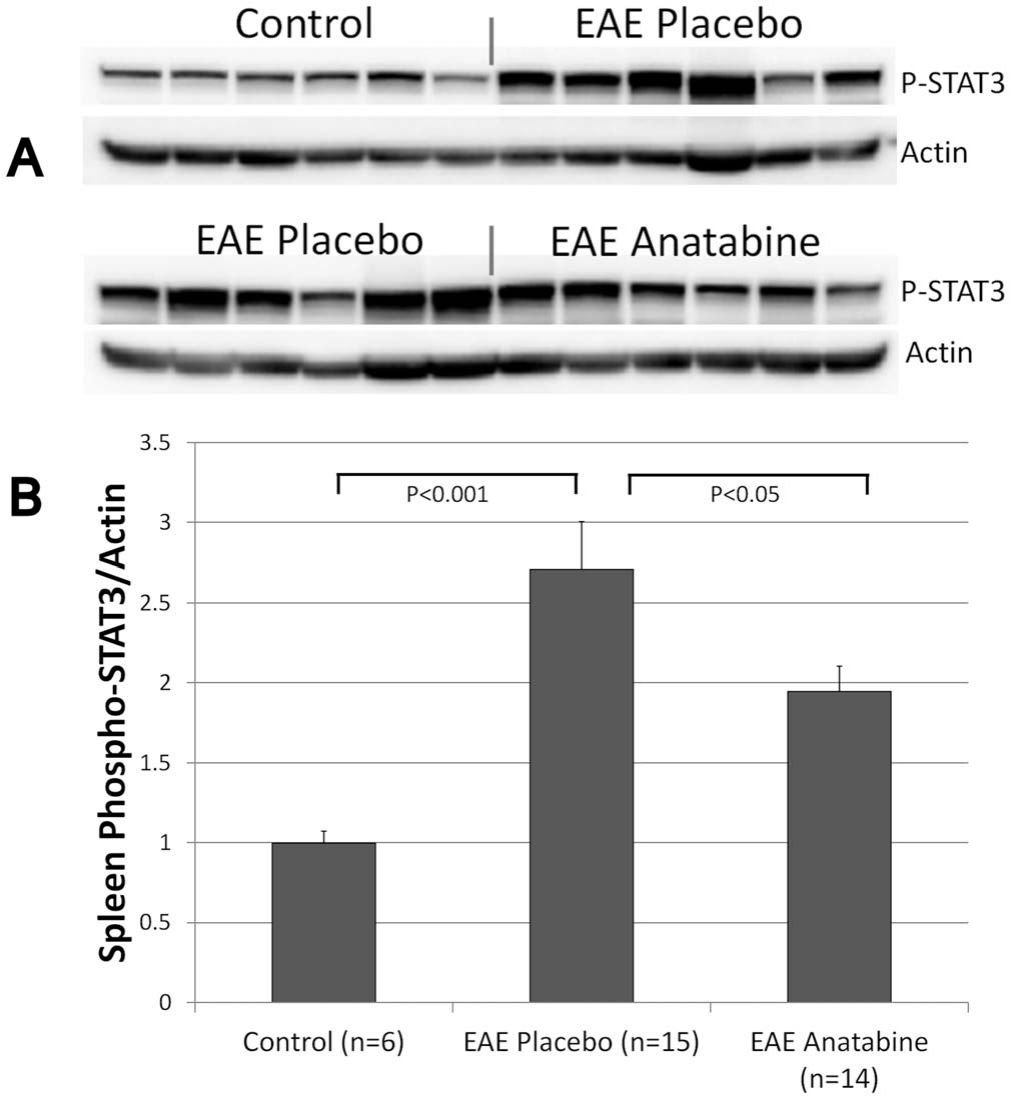
Anatabine inhibits STAT3 phosphorylation in the spleen of EAE mice. Representative western-blots showing the level of STAT3 phosphorylation in spleen homogenates from control, EAE placebo and EAE anatabine treated mice are shown. The histograms represents the average ratio of phosphorylated STAT3/Actin observed in control, EAE placebo and EAE anatabine treated mice. ANOVA reveals a statistically significant main effect of EAE (P<0.001) and of anatabine (P<0.003) on STAT3 phosphorylation levels in the spleen. Post-hoc comparisons show statistically significant differences in the amount of spleen STAT3 phosphorylation between control and EAE placebo mice (P<0.001) and between EAE placebo and EAE anatabine treated mice (P = 0.045).

**Figure 4:**
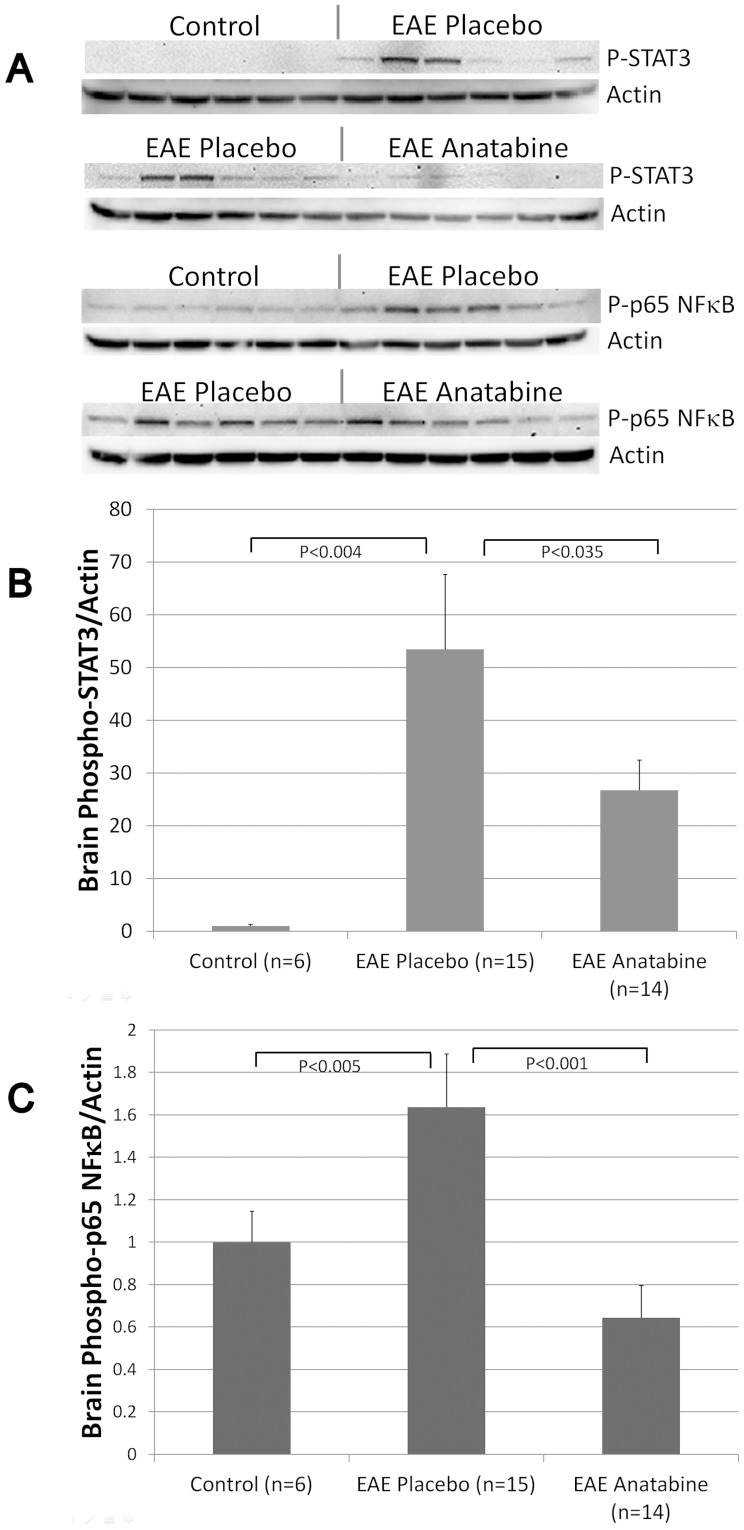
Anatabine prevents STAT3 and p65 NFκB phosphorylation in the brain of EAE mice. A) Representative western-blots depicting STAT3 phosphorylation levels in brain homogenates from control, EAE placebo and EAE anatabine treated mice are shown. B) Histogram showing the average STAT3 phosphorylation levels observed in the brain of control, EAE placebo and EAE anatabine treated mice. ANOVA reveals a statistically significant main effect of EAE (P<0.003) and of anatabine (P = 0.02) on brain STAT3 phosphorylation levels. Post-hoc comparisons show statistically significant differences in brain STAT3 phosphorylation levels between control and EAE placebo mice (P<0.004) and between EAE placebo and EAE anatabine treated mice (P<0.035). C) Histogram representing the average p65 NFκB phosphorylation levels observed in the brain of control, EAE placebo and EAE anatabine treated mice. ANOVA shows a statistically significant main effect of EAE (P<0.003) and anatabine (P<0.001) on brain p65 NFκB phosphorylation levels. Post-hoc comparisons reveal statistically significant differences in brain p65 NFκB phosphorylation levels between control and EAE placebo mice (P<0.005) and between EAE placebo and EAE anatabine treated mice (P<0.001).

**Figure 5:**
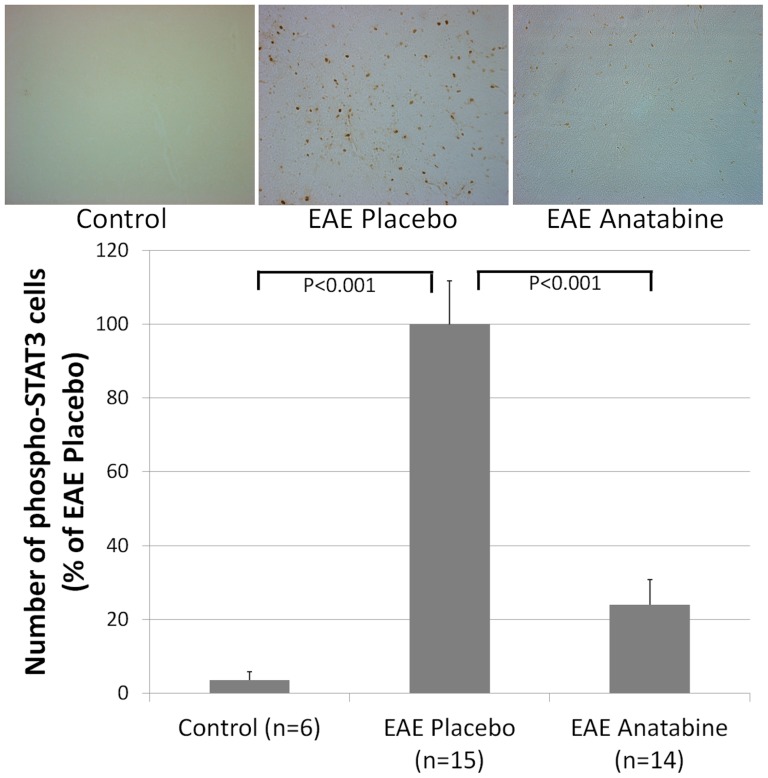
Anatabine reduces the amount of phosphorylated STAT3 immunopositive cells in the cortex of EAE mice. Representative 40X microscopic fields revealing phosphorylated STAT3 immunopositive cells in the cortex of control, EAE placebo and EAE anatabine treated mice are shown. The histogram represents the average number of phosphorylated STAT3 immunopositive cells observed in the cortex of control, EAE placebo and EAE anatabine treated mice. Results were expressed as a percentage of phospho-STAT3 immunopositive cells observed in EAE placebo mice. ANOVA reveals a statistically significant main effect of EAE (P<0.001) and of anatabine (P<0.001) on the number of phosphorylated STAT3 immunoreactive cells. Post-hoc comparisons shows statistically significant differences in the number of phospho-STAT3 immunopositive cells between control and EAE placebo mice (P<0.001) and between EAE placebo and EAE anatabine treated mice (P<0.001).

**Figure 6:**
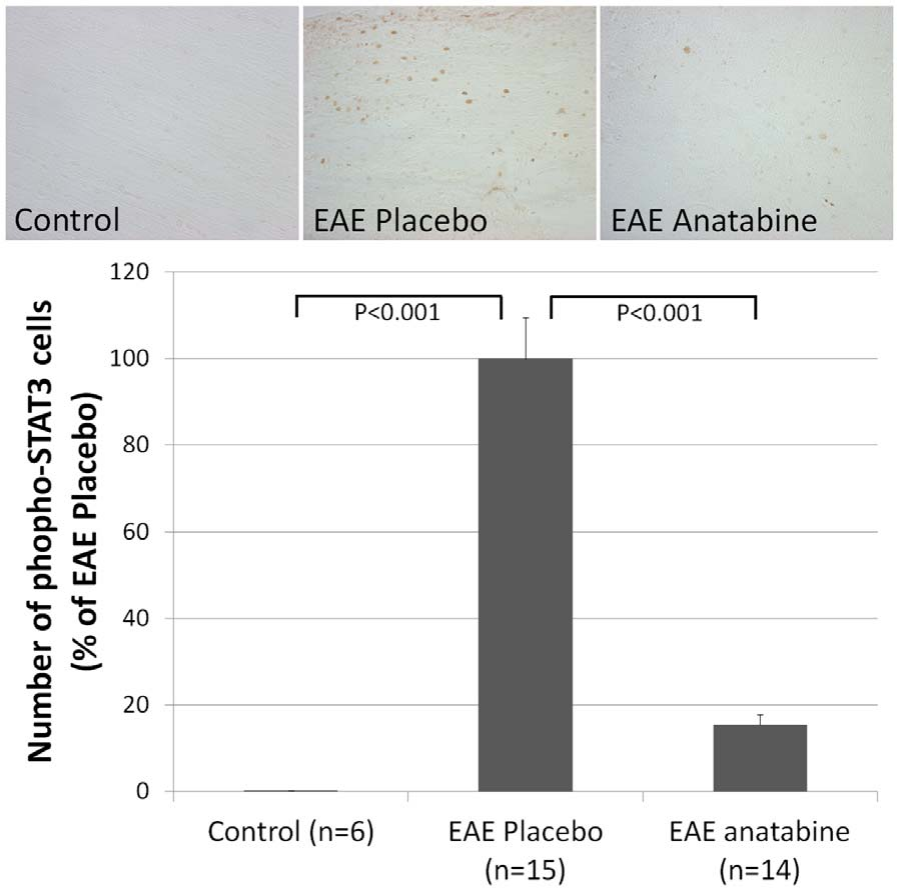
Anatabine reduces the amount of phosphorylated STAT3 immunopositive cells in the spinal cord of EAE mice. Representative 40X microscopic fields revealing phosphorylated STAT3 immunopositive cells in the spinal cord of control, EAE placebo and EAE anatabine treated mice are shown. The histogram represents the average number of phosphorylated STAT3 immunopositive cells observed in the spinal cord of control, EAE placebo and EAE anatabine treated mice. Results were expressed as a percentage of phospho-STAT3 immunopositive cells observed in EAE placebo mice. ANOVA reveals a statistically significant main effect of EAE (P<0.001) and of anatabine (P<0.001) on the number of phosphorylated STAT3 immunoreactive cells. Post-hoc comparisons shows statistically significant differences in the number of phospho-STAT3 immunopositive cells in the spinal cord between control and EAE placebo mice (P<0.001) and between EAE placebo and EAE anatabine treated mice (P<0.001).

We have shown previously that anatabine prevents STAT3 and p65 NFκB phosphorylation in various cell types and suggested that anatabine impact cytokine production by this mechanism [7] as both STAT3 and p65 NFκB are known to regulate cytokine production. We therefore measured STAT3 and p65 NFκB phosphorylation levels in EAE and EAE anatabine treated mice. Interestingly, a significant increase in STAT3 phosphorylation was observed in the spleen of EAE mice compared to control non immunized mice and a significant decrease in STAT3 phosphorylation level was detected in EAE mice treated with anatabine (Fig. 3). Western-blot analyses did not reveal a significant impact of EAE on p65 NFκB phosphorylation at day 16 post MOG immunization in the spleen (data not shown). Western-blot experiments using brain homogenates from EAE mice also reveal that both STAT3 and p65 NFκB phosphorylation are significantly elevated in the brain of EAE mice compared to control non immunized mice (Fig. 4). Additionally, we observed that anatabine significantly prevented STAT3 and p65 NFκB phosphorylation in brain homogenates of EAE mice (Fig. 4). Interestingly, a statistically significant correlation was observed between the amount of STAT3 phosphorylation detected in brain homogenates and the clinical severity of EAE (Pearson correlation  = 0.653, P<0.001) but not with p65 NFκB phosphorylation (Pearson correlation  = 0.371, P = 0.062) suggesting that STAT3 may play a more preponderant role than NFκB in the development of neurological deficits of EAE. In addition, we observed a statistically significant correlation between the amount of p65 NFκB phosphorylation and STAT3 phosphorylation in brain homogenates (Pearson Correlation = 0.637, P<0.001). For these reasons, we essentially focused on the impact of EAE and anatabine on STAT3 phosphorylation rather than on p65 NFκB phosphorylation for the remaining analyses. We further assessed STAT3 phosphorylation by immunohistochemistry using brain sections of the animals and observed an increased number of phosphorylated STAT3 immunopositive cells in the cortex of EAE mice compared to control non-immunized mice (Fig. 5). A reduction in the number of phosphorylated STAT3 immunoreactive cells was observed in the cerebral cortex of EAE mice treated with anatabine further confirming the data obtained by western-blotting using brain homogenates (Fig. 5). Similarly, we observed an increased number of phosphorylated STAT3 immunopositive cells in the spinal cord of EAE mice compared to control non-immunized mice and a decreased number of phosphorylated STAT3 immunoreactive cells in EAE mice treated with anatabine (Fig. 6) showing overall that anatabine reduces STAT3 phosphorylation in the brain, spinal cord and spleen of EAE mice. A statistically significant correlation was observed between the number of STAT3 phosphorylated cells in the cerebral cortex and the clinical severity of EAE (Pearson Correlation = 0.531, P<0.002). A statistically significant correlation was also observed between the amount of STAT3 phosphorylated cells in the spinal cord and the clinical severity of EAE (Pearson Correlation = 0.512, P<0.004).

### Effect of anatabine on pathological lesions of EAE

**Figure 7:**
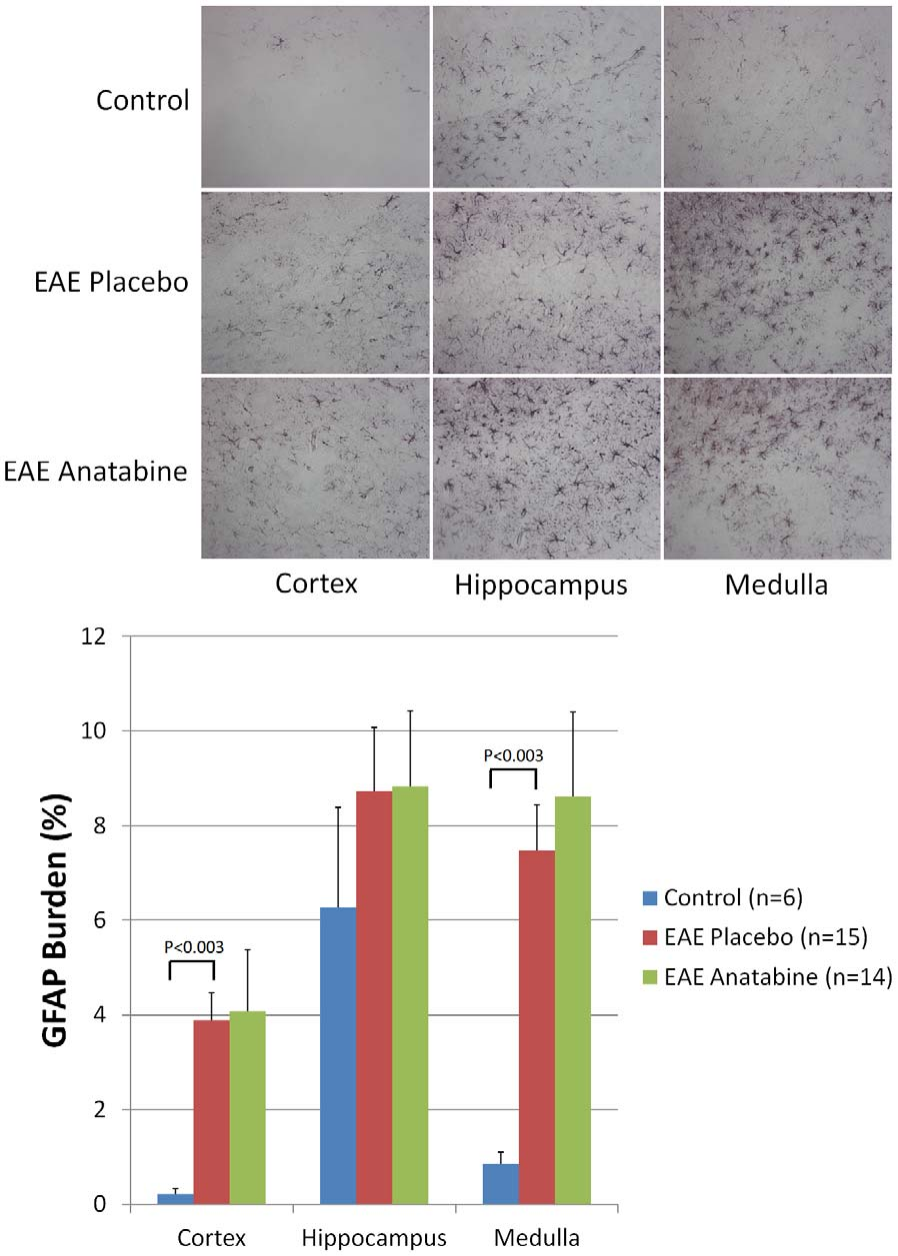
Anatabine does not affect brain astrogliosis in EAE mice. Representative 40X microscopic fields revealing GFAP immunopositive astrocytes in the cortex, hippocampus and medulla of control, EAE placebo and EAE anatabine treated mice are shown. The histogram represents the average amount of GFAP burden observed in the cortex, hippocampus and medulla of control, EAE placebo and EAE anatabine treated mice. ANOVA shows statistically significant main effect of EAE (P<0.002) and of the area of the brain examined (P<0.002) but no significant main effect of anatabine (P = 0.646) on GFAP burden. Post-hoc analyses reveal statistically significant differences in GFAP burden in the cortex between EAE placebo and control mice (P<0.003) and in the medulla between EAE placebo and control mice (P<0.003).

**Figure 8:**
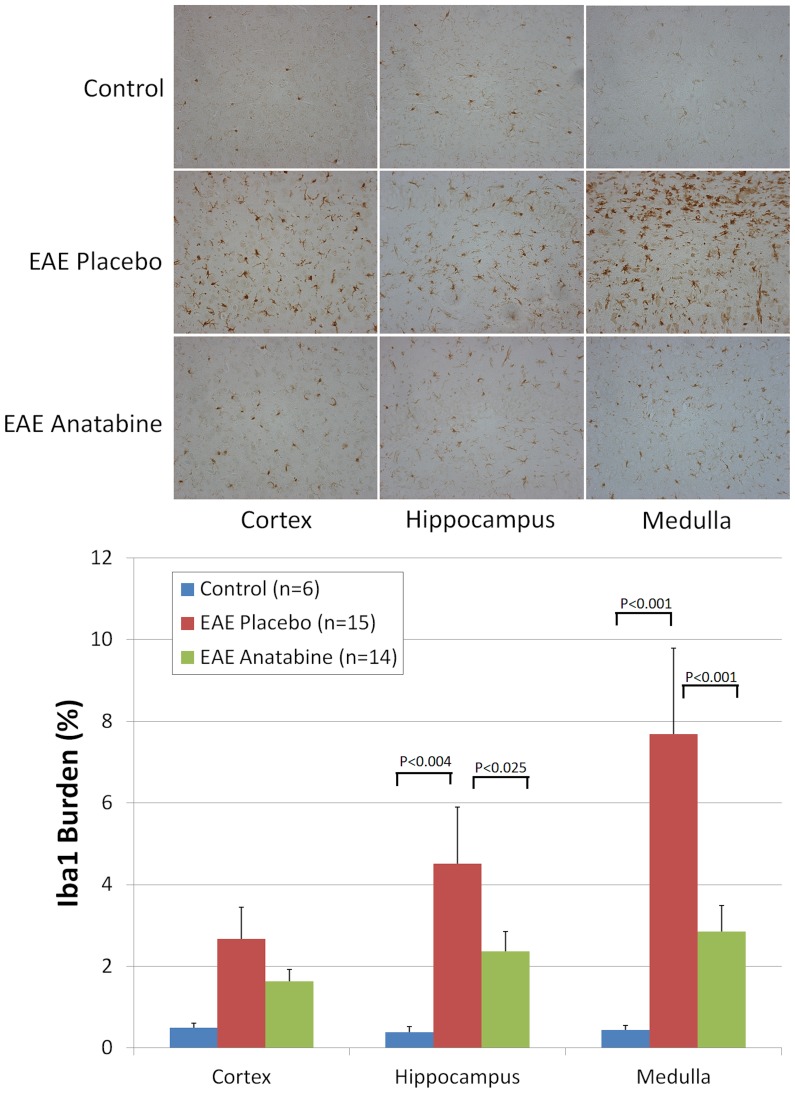
Anatabine reduces microgliosis in the brain of EAE mice. Representative 40X microscopic fields revealing Iba1 immunopositive microglial cells in the cortex, hippocampus and medulla of control, EAE placebo and EAE anatabine treated mice are shown. The histogram represents the average amount of Iba1 burden observed in the cortex, hippocampus and medulla of control, EAE placebo and EAE anatabine treated mice. ANOVA shows statistically significant main effect of EAE (P<0.001) and of anatabine (P = 0.001) on Iba1 burden. Post-hoc analyses reveal statistically significant differences in Iba1 burden in the hippocampus and medulla between EAE placebo and control mice (P<0.005) and between EAE placebo and EAE anatabine treated mice (P<0.025). No statistically significant difference in Iba1 burden was observed in the cortex between control and EAE placebo mice (P = 0.132).

As mentioned earlier, pathological lesions of EAE are not limited to the spinal cord but also occur in the brain of EAE mice. We therefore investigated the presence of microgliosis and astrogliosis in the brain of EAE mice. A significant increase in astrogliosis revealed by a GFAP immunostaining was observed in the cortex and medulla of EAE mice compared to control non immunized animals (Fig. 7). We did not observe a significant induction of astrogliosis in the hippocampus of EAE mice compared to control non-immunized animals (Fig. 7). Overall, anatabine did not significantly impact EAE induced astrogliosis for the different areas of the brain examined (Fig. 7). A significant increase in the number of microglial cells immunoreactive for Iba1 was observed in the hippocampus and medulla of EAE mice compared to control non-immunized mice (Fig. 8). Interestingly, a significant reduction in the number of IBa1 immunopositive microglial cells was found in the hippocampus and medulla of anatabine treated EAE mice (Fig. 8). There was also a trend in the same direction for microglia in the cortex but this did not reach statistical significance. Microglial cells detected in the brain of control non-immunized mice appear faintly immunostained for Iba1 and are well ramified corresponding to a healthy microglial phenotype. By comparison, microglial cells are strongly immunopositive for Iba1 in EAE brains, exhibit less ramification and appear amoeboid indicative of activated microglia (Fig. 8).

**Figure 9:**
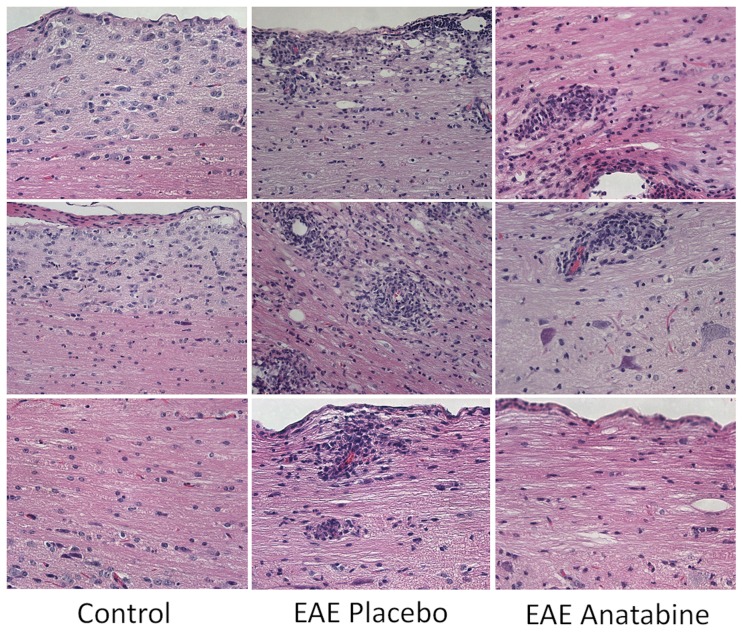
Anatabine reduces mononuclear cell infiltration in the spinal cord of EAE mice. Representative 40X microscopic fields of longitudinal sections of the spinal cord stained with hematoxylin and eosin. Intense mononuclear inflammatory infiltration of the peripheral white matter in the spinal cord of EAE placebo mice is evident compared to control mice.

**Figure 10:**
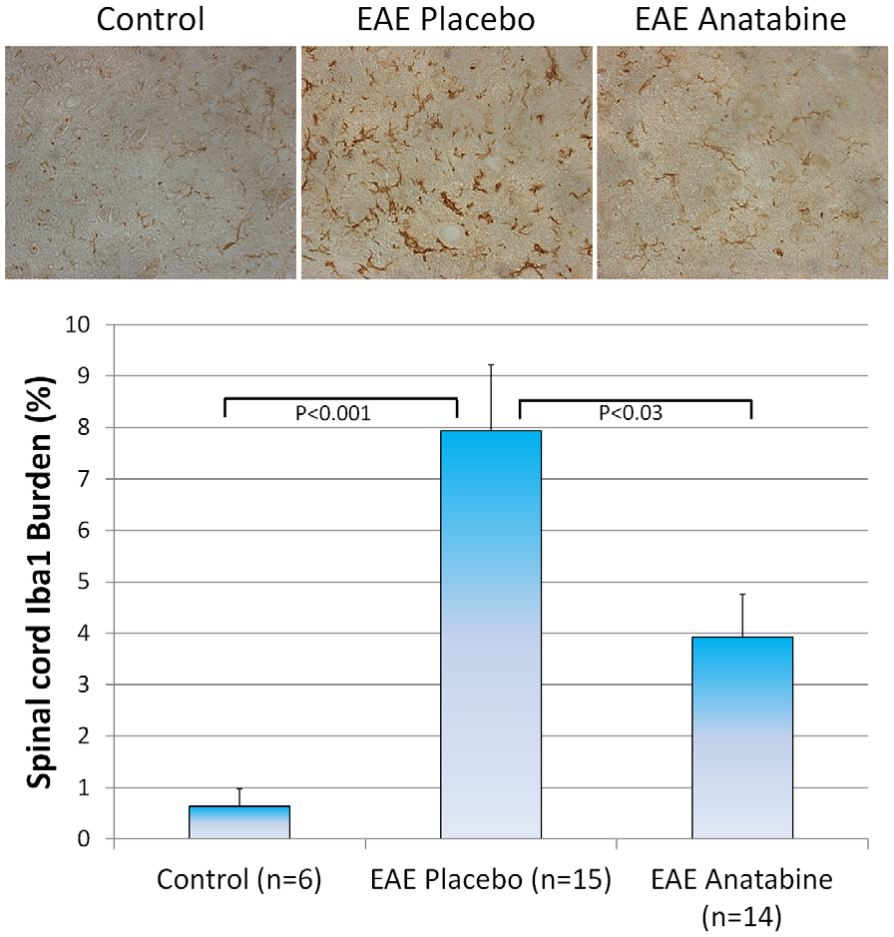
Anatabine reduces Iba1 burden in the spinal cord of EAE mice. Representative 40X microscopic fields of longitudinal sections of the spinal cord from control, EAE placebo and EAE anatabine treated mice immunostained with the microglia/macrophage marker Iba1 are shown. The histogram depicts the amount of Iba1 burden observed in the spinal cord of control, EAE placebo and EAE anatabine treated mice. ANOVA reveals a significant main effect of EAE (P<0.001) and of anatabine (P<0.009) on Iba1 burden in the spinal cord. Post-hoc comparisons show statistically significant differences for the average Iba1 burden between control and EAE placebo mice (P<0.001) and between EAE placebo and EAE anatabine treated mice (P = 0.025).

**Figure 11:**
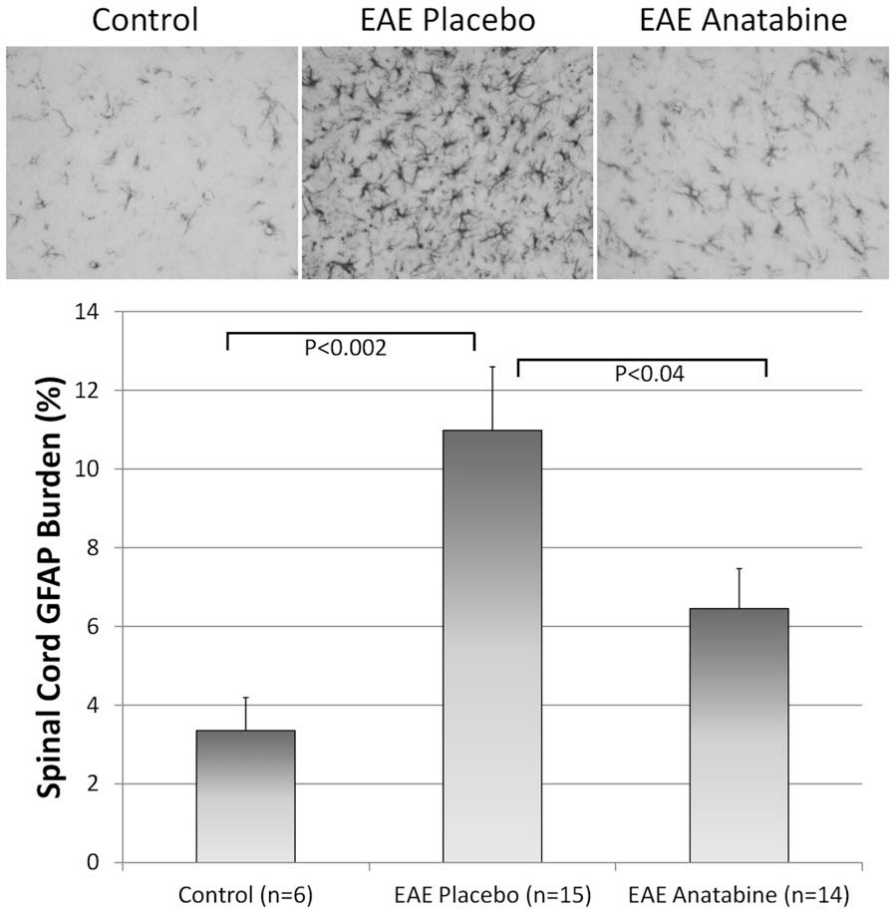
Anatabine reduces the astrogliosis in the spinal cord of EAE mice. Representative 40X microscopic fields of longitudinal sections of the spinal cord from control, EAE placebo and EAE anatabine treated mice immunostained with an anti-GFAP antibody are shown. The histogram depicts the average GFAP burden observed in the spinal cord of control, EAE placebo and EAE anatabine treated mice. ANOVA reveals a significant main effect of EAE (P = 0.002) and of anatabine (P = 0.026) on GFAP burden in the spinal cord. Post-hoc comparisons show statistically significant differences for the average GFAP burden between control and EAE placebo mice (P = 0.001) and between EAE placebo and EAE anatabine treated mice (P = 0.039).

**Figure 12:**
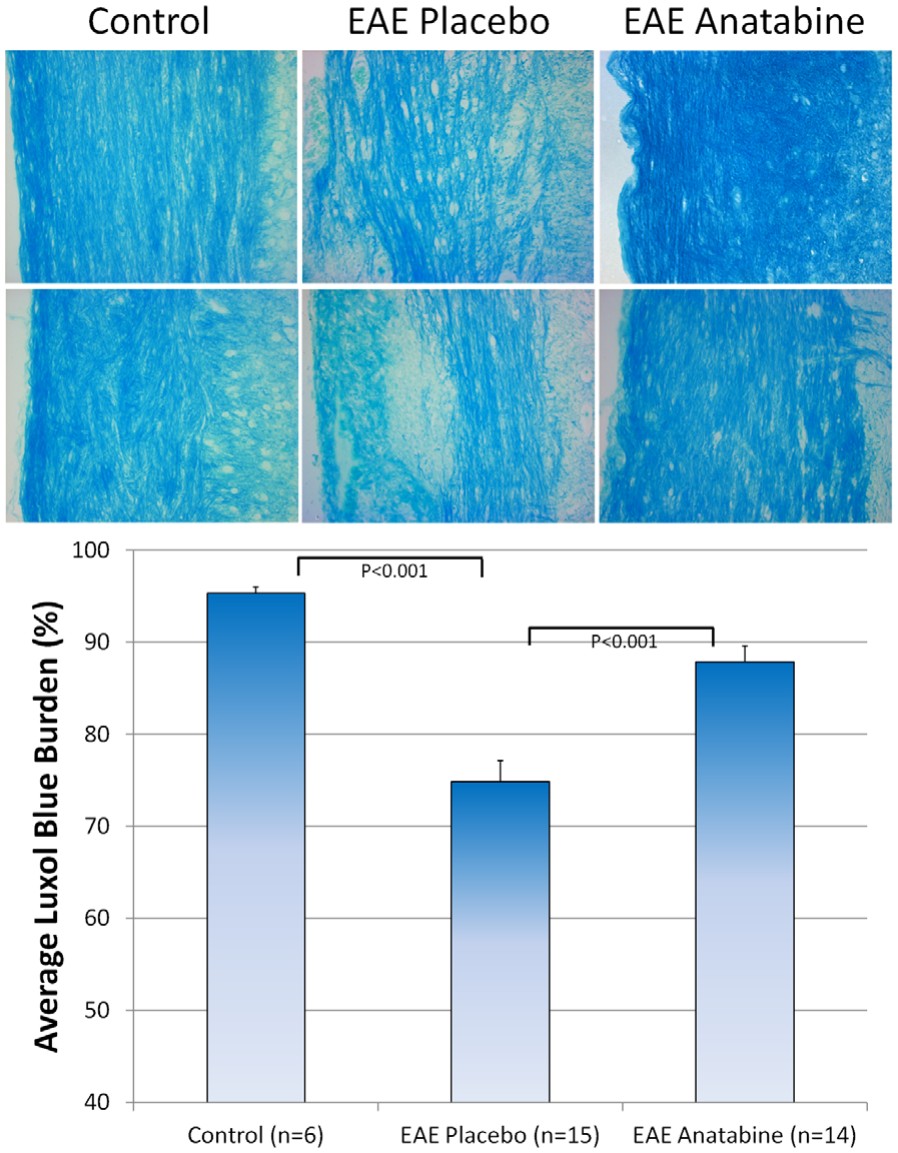
Anatabine prevents demyelination in the spinal cord of EAE mice. Representative 40X microscopic fields of longitudinal sections of the spinal cord from control, EAE placebo and EAE anatabine treated mice stained with Luxol fast blue are shown. The histogram represents the average Luxol fast blue burden observed in the spinal cord of control, EAE placebo and EAE anatabine treated mice. ANOVA reveals a significant main effect of EAE (P<0.001) and of anatabine (P<0.001) on Luxol fast blue burden in the spinal cord. Post-hoc comparisons show statistically significant differences for the average Luxol fast blue burden between control and EAE placebo mice (P<0.001) and between EAE placebo and EAE anatabine treated mice (P<0.001).

Longitudinal sections of the spinal cord from each mice were examined for the infiltration of mononuclear cells. As shown in Fig. 9, hematoxylin and eosin staining revealed numerous perivascular clusters of mononuclear infiltrating cells in the spinal cord of EAE mice and an absence of these inflammatory cell infiltrates in the spinal cord of control non-immunized mice. Less cellular infiltration was observed in the spinal cord of anatabine treated mice compared to EAE placebo mice (Fig. 9). This was further confirmed following an Iba1 immunostaining revealing the presence of Iba1 immunopositive cells in these inflammatory infiltrates. Globally, Iba1 immunostaining is significantly increased in the spinal cord of EAE mice compared to control non-immunized mice (Fig. 10). In addition, anatabine markedly decreased the Iba1 immunostaining in the spinal cord of EAE mice showing that anatabine reduces the infiltration of macrophage/microglia in the spinal cord of EAE mice (Fig. 10). A marked increase in GFAP immunostaining was also observed in the spinal cord of EAE mice and was significantly suppressed by the anatabine treatment showing that anatabine prevents astrogliosis in the spinal cord of EAE mice (Fig. 11). Finally, Luxol Fast Blue staining showed significant myelin loss in the white matter of the spinal cord of EAE mice compared control non-immunized mice (Fig. 12). These areas of demyelination appear to be located within the vicinity of perivascular inflammatory cell infiltrates. Interestingly, anatabine significantly prevented demyelination associated with EAE in the spinal cord (Fig. 12). A statistically significant positive correlation was observed between the amount of GFAP and Iba1 burden in the spinal cord (Pearson correlation = 0.612, P<0.002) whereas negative correlations were observed between the GFAP burden and the Luxol fast blue burden (Pearson correlation = −0.525, P = 0.007), and between the IBa1 burden and the Luxol fast blue burden (Pearson correlation = −0.609, P = 0.001) suggesting that astrogliosis and microgliosis are associated with the loss of myelin in the spinal cord.

## Discussion

The current study investigated anatabine as a novel treatment for MS using EAE, a well established model of MS. We found that oral administration of anatabine significantly delayed the onset of EAE and markedly decreased the severity of neurological deficits associated with EAE. EAE is mediated principally by CD4+ T lymphocytes which have been traditionally classified in function of their ability to produce different cytokines. Th1 cells are characterized by the production of INF-γ and TNF-α and have been shown to exacerbate MS by increasing inflammation [14]–[16]. Th2 cells are characterized by the production of IL-4, IL-5, IL-10 and IL-13 and exert a protective role against MS by opposing inflammation. More recently, Th17 cells which produce IL-17A, IL-17F, IL-6, IL-21, IL-22 and IL-23 have been shown to contribute to the development of MS [17] and are believed to be the main driver of autoimmune tissue injury. For instance, IL-17 deficient mice exhibit decreased severity of EAE [18] and Th17 cells are elevated in MS patients [19]. IL-6 has been shown to promote EAE pathology by stimulating the activation of microglia and astrocytes [20] and also by promoting Th17 cells differentiation and pathogenicity [21]. Our study indicates that anatabine significantly reduces IL-17A, IL-6 and IL-1β levels in the serum of EAE mice and mitigates INF-γ and IL-1β induction in the spleen of EAE mice but does not appear to affect IL-10 levels. Our data therefore suggest that anatabine inhibits pro-inflammatory Th1 and Th17 cytokines which are known to play critical roles in the induction and severity of MS and EAE.

We have shown previously that the anti-inflammatory activity of anatabine is driven by an inhibition of the transcription factors NFκB and STAT3 which control the expression of a wide variety of genes involved in inflammation including cytokines [6], [7]. For that reason, we asked whether anatabine was modulating STAT3 and p65 NFκB phosphorylation in the spleen and CNS of EAE mice. We observed that STAT3 phosphorylation was elevated in the spleen of EAE mice compared to control naive mice whereas anatabine significantly suppressed STAT3 phosphorylation. We however did not detect a significant elevation of p65 NFκB phosphorylation in the spleen of EAE mice (data not shown). In the CNS of EAE mice, we observed a stimulation of both STAT3 and p65 NFκB phosphorylation which was significantly inhibited by the anatabine treatment. Interestingly, we found a significant correlation between the amount of STAT3 phosphorylation in the CNS and the clinical severity of EAE but not with p65 NFκB phosphorylation suggesting that STAT3 activation may play a critical role in the appearance of neurological deficits associated with EAE. Interestingly, STAT3 signaling has been shown to constitute an absolute requirement for pathologic Th17 differentiation and the development of Th17-dependent autoimmunity [22]. Enhanced infiltration of inflammatory cells, increased demyelination and exacerbated clinical severity of EAE have been observed in SOC3 deficient mice displaying enhanced STAT3 signaling [23] and STAT3 knock-out mice have been shown to be resistant to EAE [22], further underscoring the requirement of STAT3 activation in CNS inflammatory diseases.

Demyelination in EAE as in MS is a result of inflammatory lesions in the white matter responsible for clinical deficits [24]. Histological analyzes of the spinal cord reveal the presence of numerous perivascular mononuclear cell infiltrates in EAE mice. These inflammatory sites are associated with a significant increase in Iba1 and GFAP immunostaining showing significant microgliosis, macrophages infiltration and astrogliosis in the spinal cord of EAE mice. Overall anatabine significantly reduces astrogliosis, microgliosis and macrophage infiltration in the spinal cord of EAE mice. Luxol fast blue staining reveals areas of demyelination associated with these inflammatory infiltrates in the white matter of EAE mice and anatabine also significantly inhibited demyelination in EAE mice suggesting a correlation with lower clinical signs.

Anatabine displays a chemical structure closely related to nicotine suggesting that it could exert its activity by stimulating nicotinic acetylcholine receptors (nAChRs). Indeed, nicotine has been shown to attenuate inflammatory and autoimmune responses in EAE mice resulting in a decreased demyelination and axonal degeneration, a delayed disease onset and an attenuation of EAE clinical severity [25]. This is consistent with the fact that the beneficial effect of nicotine against EAE has been shown to be mediated via a stimulation of nAChRs leading to a suppression of Th1 and Th17 responses [26]. Overall nicotine effects on EAE mice closely resemble those that we observed in anatabine treated EAE mice suggesting that anatabine may also mediate its beneficial activity via a stimulation of nAChRs. This is also further substantiated by the fact that stimulation of nAChRs signaling results in a suppression of STAT3 and NFκB activation [26]–[29] which we also observed following treatment with anatabine. Although nicotine has been shown to attenuate inflammation in both obesity and ulcerative colitis, its usage in the clinic has been limited due to toxicity related side effects. Extensive structure-toxicity relationships have been established previously for nicotinoids [30] and revealed that the 3-pyridylmethylamine moiety, a common structural part of most natural nicotinoids including nicotine itself is essential. Interestingly, anatabine does not possess a 3-pyridylmethylamine moiety suggesting it should be less toxic than nicotine. Anatabine may therefore offer a safer alternative to nicotine for the treatment of neuro-inflammatory related illnesses.

Overall our data show that anatabine given as a prophylactic regimen starting from the first day of the immunization exerts a beneficial effect against the acute phase of EAE. Future studies will be needed to investigate whether anatabine is capable of suppressing established EAE in order to determine whether anatabine can represent a suitable compound for the treatment of MS. Meanwhile, given that bioequivalent plasma levels of anatabine can be reached with oral doses in humans, the present data suggest that anatabine might mitigate active disease states in MS and should be piloted in clinical studies.
